# Rehmannioside A alleviates oxidative stress and restores dopaminergic function via the PI3K/Akt pathway in a rotenone-induced Parkinson’s disease model

**DOI:** 10.3389/fneur.2026.1951721

**Published:** 2026-10-20

**Authors:** Yanqi He

**Affiliations:** Mental and Health College, Qiqihar Medical University, Qiqihar, Heilongjiang, China

**Keywords:** dopaminergic system, oxidative stress, Parkinson’s disease, PI3K/Akt pathway, Rehmannioside A

## Abstract

**Objective:**

Parkinson’s disease (PD) is a progressive neurodegenerative disorder; available treatments merely relieve symptoms, and no disease-modifying therapies are currently available. This study sought to explore the neuroprotective effects of Rehmannioside A (Re A) in rotenone-induced PD models and clarify its underlying mechanisms.

**Methods:**

A rotenone-induced PD rat model was established for in vivo experiments, with rats administered graded oral doses of Re A. Motor function was assessed via behavioral tests. Striatal oxidative stress markers and neurotransmitters were detected using biochemical assays and ELISA, respectively. HE staining was applied to observe histopathological alterations in the substantia nigra, and immunohistochemistry was used to visualize tyrosine hydroxylase (TH)-positive neurons. Western blotting was performed to measure TH expression and key proteins of the PI3K/Akt pathway in brain tissue. For in vitro experiments, rotenone-challenged PC12 cells were treated with different concentrations of Re A. Cell viability was tested using CCK-8 assay, oxidative stress indicators were quantified, and protein levels were analyzed by Western blot. The PI3K inhibitor LY294002 was used to validate the reliance of protective effects on the PI3K/Akt pathway.

**Results:**

In vivo, Re A significantly rescued weight loss and motor dysfunction, mitigated striatal oxidative stress, and reduced neuronal injury and pyknosis in the substantia nigra, accompanied by an increased number of TH-positive neurons. Re A restored striatal dopamine and its metabolite levels, activated the PI3K/Akt pathway, and upregulated TH protein expression. In vitro, Re A alleviated rotenone-triggered cytotoxicity and oxidative stress, reversed the suppression of PI3K/Akt signaling, and elevated TH expression. These protective effects were partially abolished by LY294002.

**Conclusion:**

Rehmannioside A exerts neuroprotective activity against rotenone-induced PD lesions, possibly via activating the PI3K/Akt signaling pathway, suppressing oxidative stress and preserving dopaminergic function. Re A may serve as a promising therapeutic candidate for PD.

## 1 Introduction

Parkinson’s disease (PD) ranks as the second most common neurodegenerative disorder after Alzheimer’s disease, affecting an estimated population of over 10 million worldwide. Its clinical presentation is characterized by core motor symptoms, including bradykinesia, rigidity, resting tremor, and postural instability, often accompanied by a spectrum of non-motor manifestations (1, 2). The primary pathological hallmarks of PD involve the progressive loss of dopaminergic neurons in the substantia nigra pars compacta and the presence of intraneuronal inclusions known as Lewy bodies, which are primarily composed of aggregated α-synuclein. These pathological changes result in impaired dopaminergic signaling and a marked reduction in dopamine levels within the striatum, ultimately leading to the observed motor deficits (3–5). While the precise etiology of PD remains incompletely understood, key features implicated in its neurodegenerative process have been identified. These include the abnormal aggregation of α-synuclein (α-syn) into cytoplasmic inclusions, mitochondrial dysfunction, elevated oxidative stress, neuroinflammation, and dysregulation of autophagy (6, 7).

The phosphatidylinositol 3-kinase/protein kinase B (PI3K/Akt) signaling pathway is a central regulator of diverse cellular processes, including growth, proliferation, migration, and metabolism, and plays a pivotal role in mitigating oxidative stress (8–10). In neurodegenerative disorders such as Parkinson’s disease, this pathway is involved not only in modulating inflammatory responses but also in mediating the protective effects of antioxidants on dopaminergic neurons (11, 12). The PI3K/Akt pathway contributes significantly to inflammatory regulation in PD (13). Its downstream substrate glycogen synthase kinase-3β (GSK-3β) can influence the survival of dopaminergic neurons through the regulation of oxidative stress (14). Another critical downstream effector, the transcription factor nuclear factor erythroid 2–related factor 2 (Nrf2), plays an essential role in activating the antioxidant response and preventing mitochondrial damage induced by oxidative stress (15). Consequently, identifying bioactive agents capable of effectively modulating the PI3K/Akt signaling pathway represents a promising direction for therapeutic intervention in PD. In this context, traditional Chinese medicines and their active components have shown potential value for such regulatory purposes.

Rehmannioside A (Re A) is a natural compound derived from the traditional Chinese herb Rehmanniae Radix (16). It exerts multi-faceted protective effects, primarily through modulation of the PI3K/Akt signaling pathway. Previous studies have demonstrated that Re A can alleviate ferroptosis and improve post-ischemic cognitive impairment by activating the PI3K/Akt/Nrf2 and SLC7A11/GPX4 axes. Furthermore, Re A exhibits neuroprotective properties in models of vascular dementia and spinal cord injury by suppressing oxidative stress and inflammatory responses (17–19). Although PI3K/Akt signaling has been implicated in neuroprotection in experimental PD models (11, 12), the role of Re A in PD remains unknown. Therefore, the present study employed rotenone-induced cellular and rat models of PD to investigate whether Rehmannioside A can ameliorate behavioral deficits, oxidative stress, and dopaminergic dysfunction. PI3K/Akt was selected as our primary candidate pathway because it plays a critical role in dopaminergic neuron survival and antioxidant defense in PD, and prior neuroprotective studies of Re A also pointed to this signaling axis. Furthermore, we preliminarily examined whether its potential protective effects are associated with the modulation of the PI3K/Akt signaling pathway.

## 2 Materials and methods

### 2.1 Animal experiments

All animal procedures and experiments were approved by the Animal Ethics Committee of Qiqihar Medical University (Protocol No. QMU-AECC-2025-237) and conducted in accordance with the ARRIVE guidelines. Eight- to 10-week-old male Sprague–Dawley rats weighing 260 ± 20 g were randomly divided into six groups (*n* = 6): Control, Model, Re A-L (low dose: 20 mg·kg^−1^), Re A-M (medium dose: 40 mg·kg^−1^), Re A-H (high dose: 80 mg·kg^−1^), and a positive control group receiving Levodopa + Carbidopa. The doses of Re A were selected based on previously reported oral administration studies in rodents, in which Re A at comparable doses did not cause observable toxicity (18, 20). To induce Parkinson’s disease, rats in all groups except the Control received a daily subcutaneous injection of rotenone (2 mg·kg^−1^, dissolved in sunflower oil) for 28 consecutive days. Rats in the Control group were injected subcutaneously with an equal volume of sunflower oil vehicle only. Re A treatment began on the same day as rotenone exposure and continued once daily for the entire 28-day modeling period. On each day, Re A was administered by oral gavage first, and rotenone was injected subcutaneously 2 h later. During model establishment, animals gradually developed behavioral abnormalities such as bradykinesia, unstable gait, and postural imbalance, which confirmed successful model induction (21, 22). Drug intervention was administered concurrently during the modeling period. Re A was given orally (by gavage) at the respective doses once daily. The Control and Model groups received an equal volume of vehicle (10% DMSO, 40% PEG300, 5% Tween-80, and 45% saline) via gavage. The positive control group was administered Levodopa + Carbidopa by oral gavage according to the standard protocol. All rats were euthanized 24 h after the last rotenone administration. Animals were divided into two cohorts: one cohort was perfused with phosphate-buffered saline (PBS) for Western blot and DA/DOPAC/HVA measurements, and the other cohort was perfused with PBS followed by 4% paraformaldehyde (PFA) for HE staining and TH immunohistochemistry.

### 2.2 Rotarod test

The rotarod test was performed to evaluate motor coordination in rats. Prior to formal testing, all animals underwent an acclimation session on the rotating rod. Each rat was habituated on the rod for 10 min, followed by a 15 min rest. The formal test consisted of three trials with an inter-trial interval of 15 min, and the mean latency to fall across the three trials was used for analysis. The formal test was conducted 24 h after the last administration. The rod rotation speed was set at 15 rpm, with a maximum test duration of 300 s. A constant-speed protocol was used as previously described (21, 23). The latency to fall from the rod was recorded for each rat.

### 2.3 Open filed test

To assess general locomotor and exploratory behavior, an open field test was conducted. The apparatus floor was virtually subdivided into a 5 × 5 grid of equal squares (each 25 cm × 25 cm). Each rat was gently placed in the central square at the start of the trial, and its movement was recorded for 5 min using a video tracking system. After testing each animal, feces were removed and the arena floor was thoroughly wiped with 75% ethanol to eliminate olfactory cues for subsequent trials.

### 2.4 Hematoxylin and eosin staining

Rats were intraperitoneally anesthetized with sodium pentobarbital. Whole brains were rapidly removed, and brain tissues containing the substantia nigra were fixed in 4% paraformaldehyde for subsequent processing. The fixed tissues were sequentially sectioned, heated, deparaffinized, and rehydrated. The sections were then stained with hematoxylin for 6 min, rinsed with 1% acid ethanol for 30 s, and counterstained with 0.5% eosin for 2 min. After dehydration, the sections were mounted with neutral resin.

### 2.5 Tyrosine hydroxylase immunohistochemistry

Brain tissues embedded in paraffin were sectioned at 4 μm, deparaffinized, rehydrated, and subjected to antigen retrieval. The sections were then incubated with TH Polyclonal antibody (25859-1-AP, Proteintech, China; 1:1000) overnight at 4 °C. After washing with PBS, the sections were incubated with SP ultra-sensitive reagent for 2 h at room temperature. Immunoreactivity was visualized using DAB, and the sections were then dehydrated, cleared, and mounted. For evaluation, sections containing the substantia nigra were selected under a microscope, and photomicrographs were taken under consistent background light intensity. The boundaries of the substantia nigra pars compacta were delineated according to a stereotaxic atlas. Three sections per animal were analyzed bilaterally, and TH-positive neurons were counted using ImageJ software. The results are expressed as the percentage of TH-positive cell.

### 2.6 Measurement of dopamine, DOPAC, and HVA

The striatal tissues were rapidly dissected, snap-frozen in liquid nitrogen, and stored at −80 °C until analysis. Striatal tissue was weighed before homogenization, and the results were normalized to wet tissue weight. For dopamine measurement, tissues were homogenized in ice-cold 0.01 N hydrochloric acid containing 1 mM EDTA and 4 mM sodium metabisulfite, followed by centrifugation to collect the supernatant. Dopamine levels were determined strictly according to the instructions of the commercial assay kit (LDN, Germany). For the measurement of 3,4-dihydroxyphenylacetic acid (DOPAC) and homovanillic acid (HVA), tissue samples were separately homogenized in ice-cold phosphate-buffered saline (PBS, pH 7.4) and centrifuged. The resulting supernatants were analyzed using corresponding ELISA kits (COIBO BIO, China) following the manufacturer’s protocols.

### 2.7 Cell culture

Rehmannioside A (Re A) was purchased from Shanghai Yuanye Bio-Technology Co., Ltd. (B20292-5 mg, purity ≥ 98% by HPLC). PC12 cells (Fuheng Biology, China) were cultured in high-glucose Dulbecco’s Modified Eagle Medium (DMEM; SiJiQing, China) supplemented with 10% fetal bovine serum (vazyme, China), 100 U/mL penicillin (Biosharp, China), and 100 μg/mL streptomycin (Biosharp, China). Cells were incubated at 37 °C in a humidified atmosphere containing 5% CO₂. The cells were divided into the following groups: Control group (complete medium only); Model group (1 μmol/L rotenone); Re A groups (pretreated with 25 (Re A-L), 50 (Re A-M), or 100 μmol/L (Re A-H) Re A for 30 min, followed by 1 μmol/L rotenone); LY294002 group (10 mM LY294002 plus 1 μmol/L rotenone); and Re A + LY294002 group (10 mM LY294002, 100 μmol/L Re A, and 1 μmol/L rotenone).

### 2.8 Cell viability assay

PC12 cells in the logarithmic growth phase were seeded into 96-well plates. After attachment, cells were treated according to the aforementioned experimental groups and incubated for 24 h. Two hours before the end of incubation, 10 μL of CCK-8 reagent (Biosharp, China) was added to each well, followed by further incubation for 2 h under standard culture conditions. The absorbance at 450 nm was measured using a microplate reader.

### 2.9 Measurement of MDA, GSH, and SOD levels

PC12 cells were seeded in 6-well plates and treated according to the designated groups. After treatment, the cell culture supernatant was collected. For tissue samples, homogenate supernatant was prepared from animal tissues. The levels of malondialdehyde (MDA), superoxide dismutase (SOD), and glutathione (GSH) were then measured strictly following the instructions provided with the respective assay kits. All kits were purchased from Nanjing Jiancheng Bioengineering Institute (China).

### 2.10 Western blot analysis

Following treatment, cells were lysed on ice for 30 min using 500 μL of RIPA lysis buffer (Biosharp, China) supplemented with 1 mmol/L PMSF. The lysates were centrifuged at 12,000 × g for 10 min at 4 °C, and the supernatants were collected. Tissue samples were individually weighed and homogenized in ice-cold RIPA buffer. The lysates were then heat-denatured at 95 °C for 10 min, followed by centrifugation at 12,000 × g for 10 min at 4 °C. Following centrifugation, the supernatant was collected for subsequent analysis. Protein concentration was determined using a BCA protein assay kit (Biosharp, China). Equal amounts of protein were separated by SDS-PAGE and transferred onto PVDF membranes (Epizyme, China). The membranes were then incubated overnight at 4 °C with the following primary antibodies: anti-phospho-PI3K (AF3242, Affinity Biosciences, China; 1:2000), anti-PI3K (25868-1-AP, Proteintech, China; 1:4000), anti-phospho-Akt (AF6261, Affinity Biosciences, China; 1:2000), anti-Akt (AF0908, Affinity Biosciences, China; 1:2000), anti-TH (25859-1-AP, Proteintech, China; 1:50000), and anti-GAPDH (AF7021, Affinity Biosciences, China; 1:30000). After washing with PBS, the membranes were incubated with an HRP-conjugated goat anti-rabbit IgG secondary antibody (SA00001-2, Proteintech, China; 1:10000) at room temperature for 1 h, followed by TBST washes. Protein bands were visualized using an ECL detection kit (Proteintech, China) and imaged. The band intensities were quantified using ImageJ software, and the relative expression of each target protein was normalized to that of GAPDH.

### 2.11 Statistical analysis

Data were analyzed using SPSS software (version 22.0) and the results were expressed as mean ± standard deviation (SD). Comparisons among multiple groups were performed by one-way analysis of variance (ANOVA), followed by Tukey’s test for post-hoc pairwise comparisons. A *p*-value of less than 0.05 was considered statistically significant.

## 3 Results

### 3.1 Re a attenuates motor deficits in a rotenone-induced rat model of Parkinson’s disease

As shown in Figure 1A, body weight in the Control group remained stable throughout the experimental period. In contrast, rats in the Model group began to lose weight from day 7 onward compared with the Control group. Administration of Re A at each dose, as well as the positive drug treatment, appeared to attenuate this declining trend in body weight relative to the model group.

**Figure 1:**
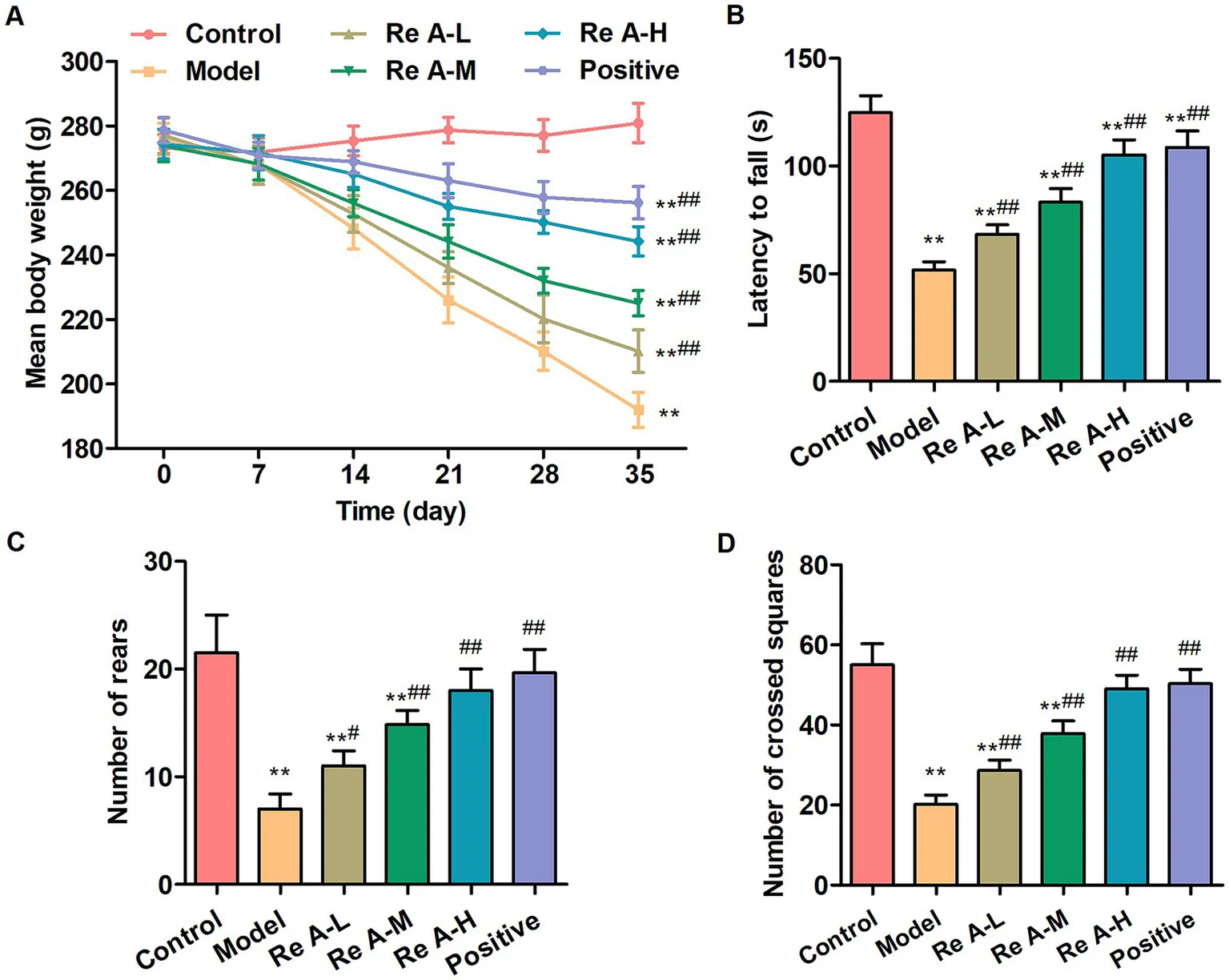
Effects of Re A on body weight and motor behavior in rotenone-induced Parkinson’s disease model rats. **(A)** Body weight changes of rats in each group during the experimental period (days 0, 7, 14, 21, 28, 35). **(B)** Rotarod test: latency to fall from an accelerating rotating rod was recorded. **(C)** Open field test: number of rearings (hind-limb standing events) within 5 min was measured. **(D)** Open field test: number of grid crossings within 5 min was recorded. Data are expressed as mean ± SD (*n* = 6 per group for *in vivo* experiments; *n* = 3 independent experiments for *in vitro* assays). * *p* < 0.05, ** *p* < 0.01 vs. Control group; # *p* < 0.05, ## *p* < 0.01 vs. Model group.

In the rotarod test (Figure 1B), the latency to fall was significantly shorter in the Model group than in the Control group (*p* < 0.05). Compared with the Model group, the low-, medium- and high-dose Re A groups, as well as the Positive group, showed a significant prolongation in fall latency (*p* < 0.05).

During the open field test (Figures 1C,D), the Model group exhibited significantly fewer rearing episodes and a shorter total distance traveled compared with the Control group(*p* < 0.05). Relative to the Model group, the low-, medium- and high-dose Re A treatments, along with the positive drug, resulted in a significant increase in both rearing frequency and total locomotion distance (*p* < 0.05). The exact numerical values and error estimates for each comparison are shown in the corresponding figures.

### 3.2 Re a attenuated rotenone-induced neuronal loss in the substantia Nigra

As shown in Figure 2A, HE staining of the substantia nigra pars compacta revealed that rotenone treatment induced marked neuronal damage and pyknosis in the Model group compared with the Control group, whereas ReA administration (20, 40, and 80 mg/kg) and the positive control visibly alleviated these histopathological changes. Furthermore, TH immunohistochemical staining (Figure 2B) showed that the percentage of TH-positive cell in the substantia nigra was significantly reduced in the Model group compared with the Control group (*p* < 0.05), while ReA treatment dose-dependently increased the percentage of TH-positive cell relative to the Model group (*p* < 0.05). The exact numerical values and error estimates for each comparison are shown in the corresponding figures.

**Figure 2:**
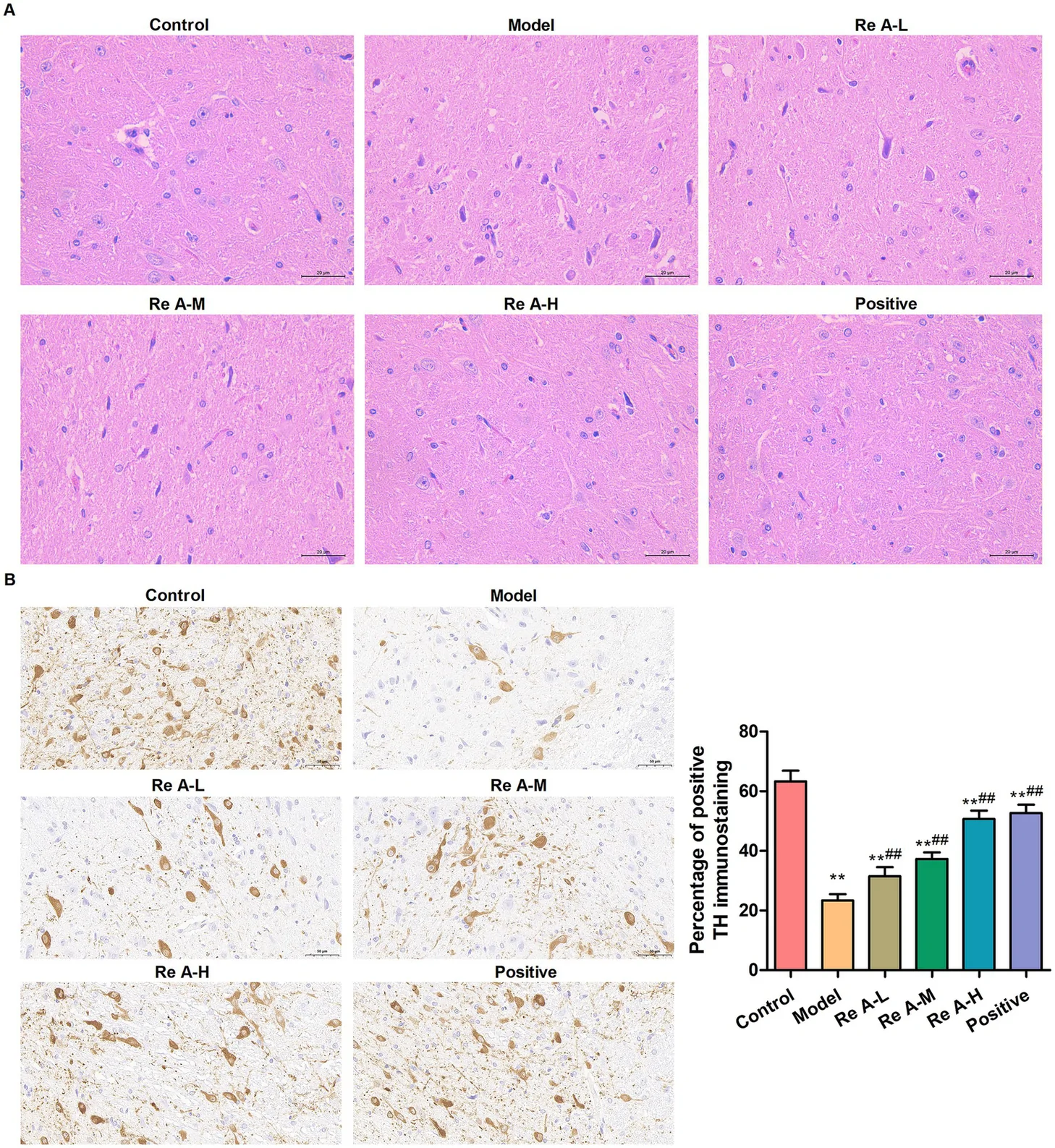
Effects of ReA on histopathological changes and TH-positive neurons in the substantia nigra pars compacta of rotenone-induced PD rats. **(A)** HE staining showing neuronal morphology (scale bar = 20 μm). **(B)** TH immunohistochemical staining and quantification of TH-positive neurons (scale bar = 50 μm). TH-positive neurons were counted in three 4-μm sections per animal bilaterally using ImageJ; values are presented as the percentage of TH-positive cell. Data are presented as mean ± SD (*n* = 6 per group for *in vivo* experiments; *n* = 3 independent experiments for *in vitro* assays). *p < 0.05, **p < 0.01 vs. Control group; #*p* < 0.05, ##*p* < 0.01 vs. Model group.

### 3.3 Re a ameliorates oxidative stress and restores dopamine levels in the striatum of PD rats

As shown in Figures 3A–C, compared with the Control group, the Model group exhibited a significant increase in MDA content and a decrease in both GSH levels and SOD activity in the striatum (*p* < 0.05). Treatment with Re A at all doses (20, 40, and 80 mg/kg) and the positive control (levodopa + carbidopa) appeared to partially reverse these alterations, reducing MDA content and increasing both GSH levels and SOD activity relative to the Model group.

**Figure 3:**
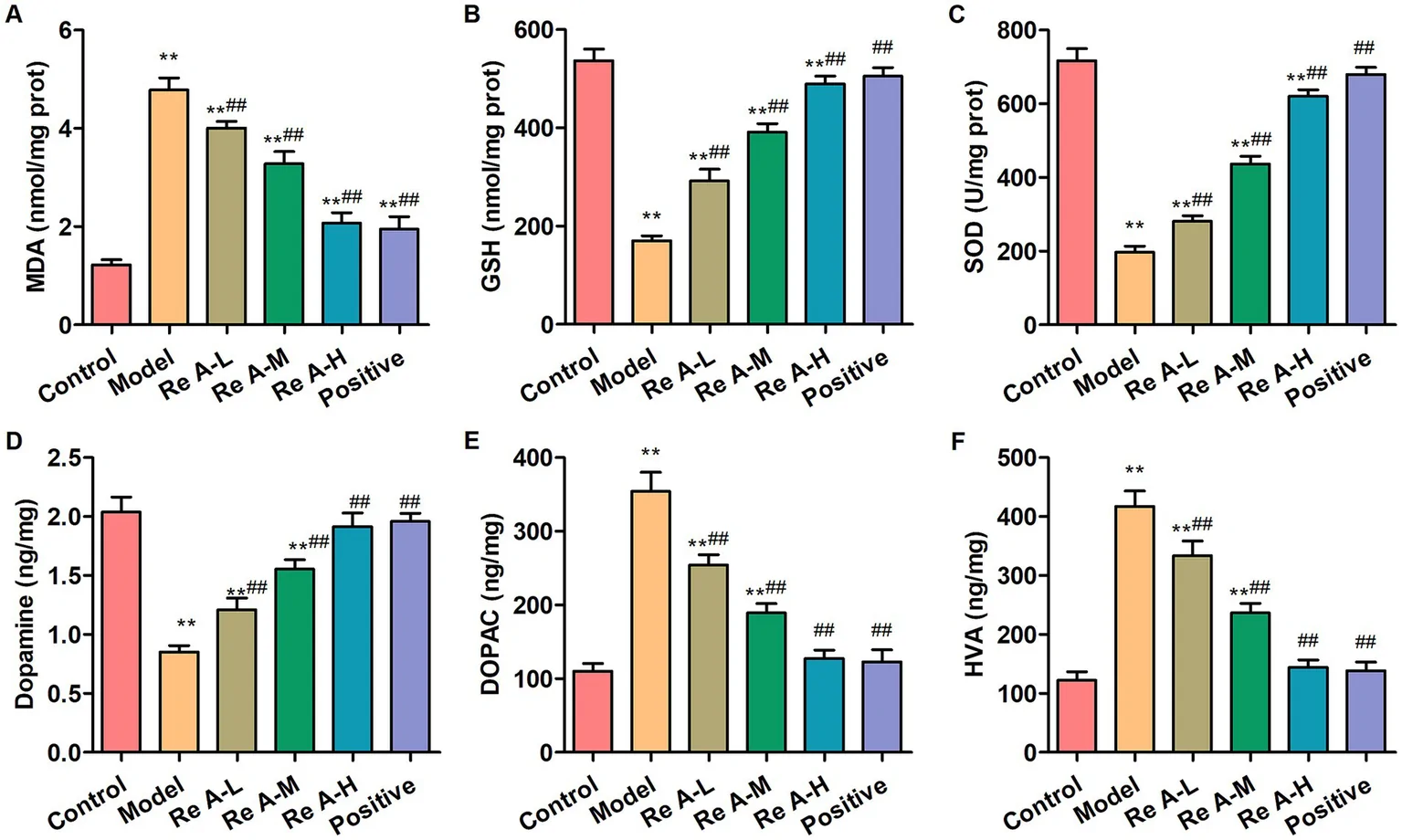
Effects of Re A on striatal oxidative stress and dopaminergic function in a rotenone-induced Parkinson’s disease rat model. Levels of MDA **(A)** and GSH **(B)**, and activity of SOD **(C)** in rat striatal tissue. Striatal concentrations of DA **(D)** and its metabolites, DOPAC **(E)** and HVA **(F)**, measured by ELISA. Values are expressed as ng per mg wet tissue weight. Data are expressed as mean ± SD (n = 6 per group for *in vivo* experiments; n = 3 independent experiments for *in vitro* assays). *p < 0.05, **p < 0.01 vs. Control group; #*p* < 0.05, ##*p* < 0.01 vs. Model group.

The status of the dopaminergic system in the striatum was further assessed by ELISA (Figures 3D–F). DA levels were significantly lower in the Model group than in the Control group, whereas the concentrations of its metabolites, DOPAC and HVA, were elevated (*p* < 0.05). Compared with the Model group, Re A treatment dose-dependently increased DA levels while reducing DOPAC and HVA concentrations. The exact numerical values and error estimates for each comparison are shown in the corresponding figures.

### 3.4 Re a is associated with enhanced PI3K/AKT signaling and concomitant TH upregulation in PD rats

As shown in Figure 4, compared with the Control group, the Model group showed significantly decreased protein levels of p-PI3K, p-Akt and TH in brain tissue (*p* < 0.05). Administration of Re A (20, 40, and 80 mg/kg) restored the levels of p-PI3K and p-Akt in a dose-dependent manner relative to the Model group. TH protein expression was similarly up-regulated following Re A treatment, with the most pronounced effect observed in the high-dose group (80 mg/kg) (*p* < 0.05). The positive control (levodopa + carbidopa) also exhibited comparable up-regulating effects on p-PI3K, p-Akt, and TH protein levels, similar to those seen in the high-dose Re A group. The exact numerical values and error estimates for each comparison are shown in the corresponding figures.

**Figure 4:**
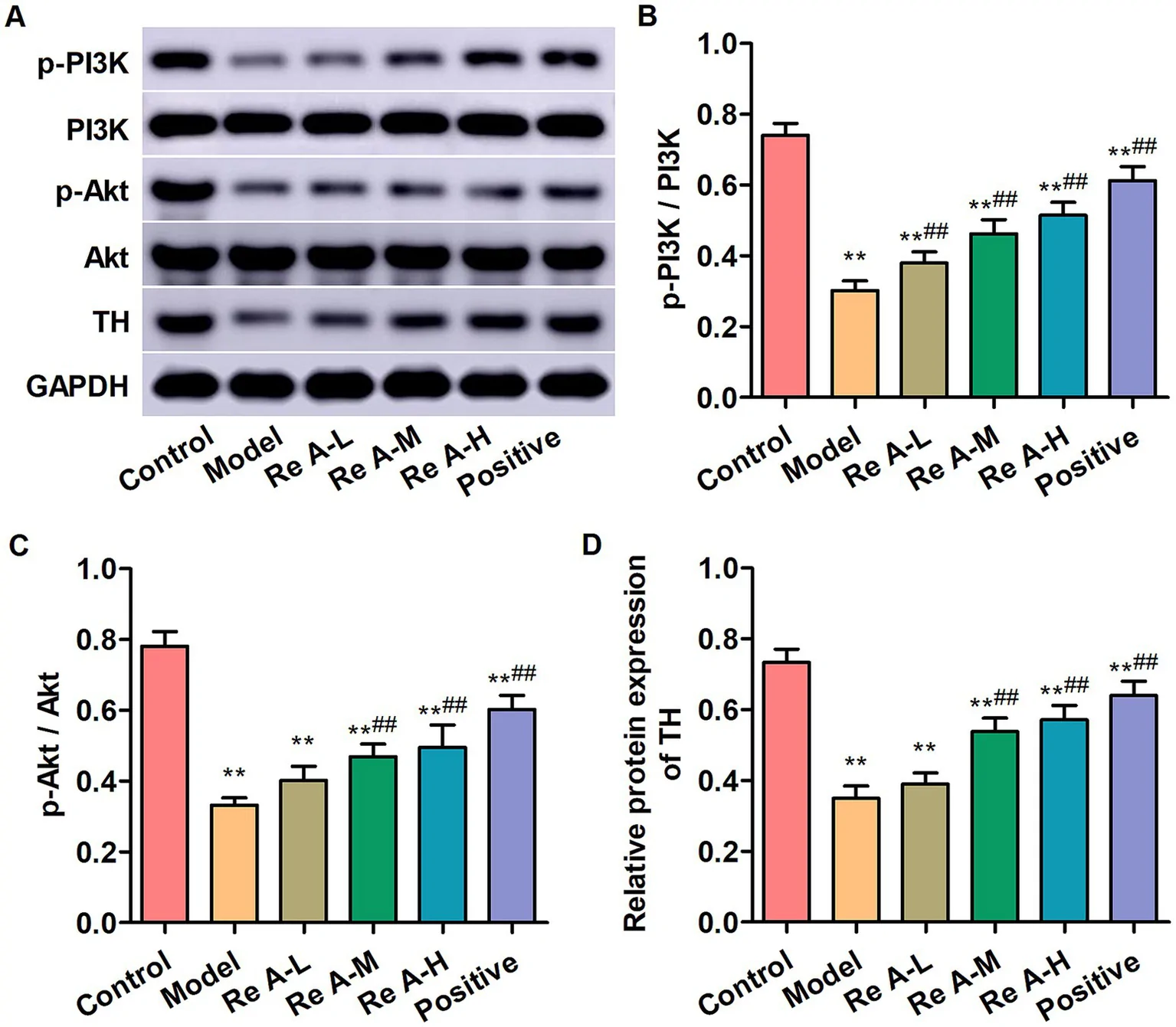
Effects of Re A on the PI3K/Akt signaling pathway and TH protein expression in brain tissues of Parkinson’s disease model rats. **(A)** Representative Western blot images showing protein levels of p-PI3K, total PI3K, p-Akt, total Akt, and TH. Quantitative analysis of the relative expression of **(B)** p-PI3K/PI3K, **(C)** p-Akt/Akt, and **(D)** TH/GAPDH. Data are presented as mean ± SD (*n* = 6 per group for *in vivo* experiments; *n* = 3 independent experiments for *in vitro* assays). * *p* < 0.05, ** *p* < 0.01 vs. Control group; # *p* < 0.05, ## *p* < 0.01 vs. Model group.

### 3.5 Re a alleviates rotenone-induced oxidative stress and restores PI3K/Akt signaling in PC12 cells

As shown in Figure 5A, exposure to 1 μM rotenone significantly reduced PC12 cell viability, while Re A pretreatment (10–200 μM) restored cell viability to varying degrees. Based on these results, three concentrations (25, 50, and 100 μM) were selected for subsequent experiments. As presented in Figure 5B, rotenone markedly increased MDA levels and decreased GSH content and SOD activity, whereas Re A pretreatment dose-dependently reversed these oxidative stress markers. Furthermore, Western blot analysis (Figure 5C) revealed that rotenone significantly downregulated p-PI3K, p-Akt, and TH protein expression, effects that were attenuated by Re A in a dose-dependent manner (*p* < 0.05). The exact numerical values and error estimates for each comparison are shown in the corresponding Figures.

**Figure 5:**
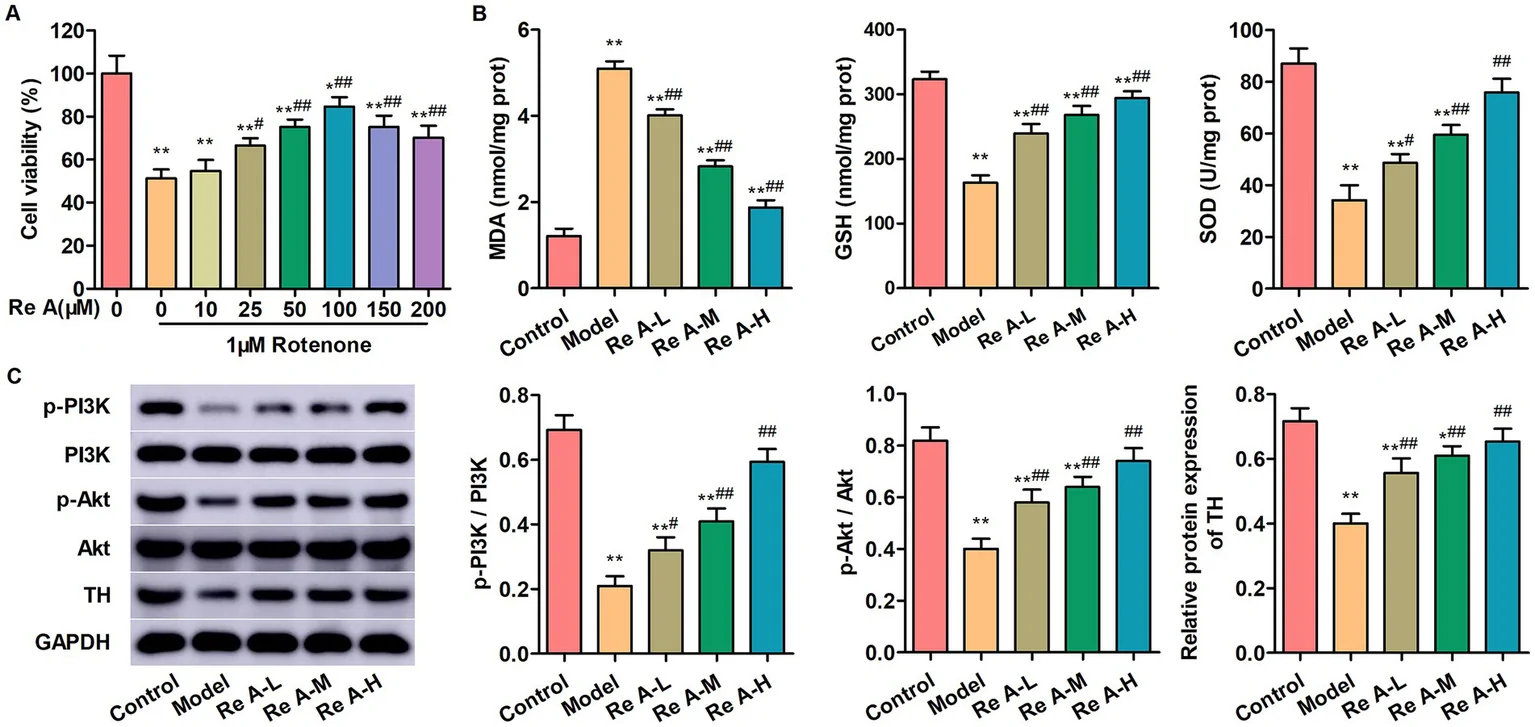
Re A attenuated rotenone-induced cytotoxicity, oxidative stress, and PI3K/Akt pathway inhibition in PC12 cells. **(A)** Cell viability assessed by CCK-8 assay. **(B)** MDA, GSH, and SOD levels. **(C)** Protein expression of p-PI3K, PI3K, p-Akt, Akt, and TH detected by Western blot. Data are presented as mean ± SD (n = 6 per group for *in vivo* experiments; n = 3 independent experiments for *in vitro* assays). *P < 0.05, **P < 0.01 vs. Control group; #*P* < 0.05, ##*P* < 0.01 vs. Model group.

### 3.6 PI3K inhibitor LY294002 reverses the protective effects of re a in PC12 cells

To further verify whether the protective effects of Re A are mediated through the PI3K/Akt pathway, the PI3K inhibitor LY294002 was used. As shown in Figure 6A, the antioxidant effects of Re A (reduced MDA, increased GSH and SOD) were partially reversed by LY294002. Meanwhile, LY294002 alone further aggravated rotenone-induced oxidative stress. Western blot analysis (Figure 6B) revealed that LY294002 attenuated the Re A-induced upregulation of p-PI3K, p-Akt, and TH expression. These results suggest that the neuroprotective effects of Re A are at least partly dependent on activation of the PI3K/Akt pathway. The exact numerical values and error estimates for each comparison are shown in the corresponding figures.

**Figure 6:**
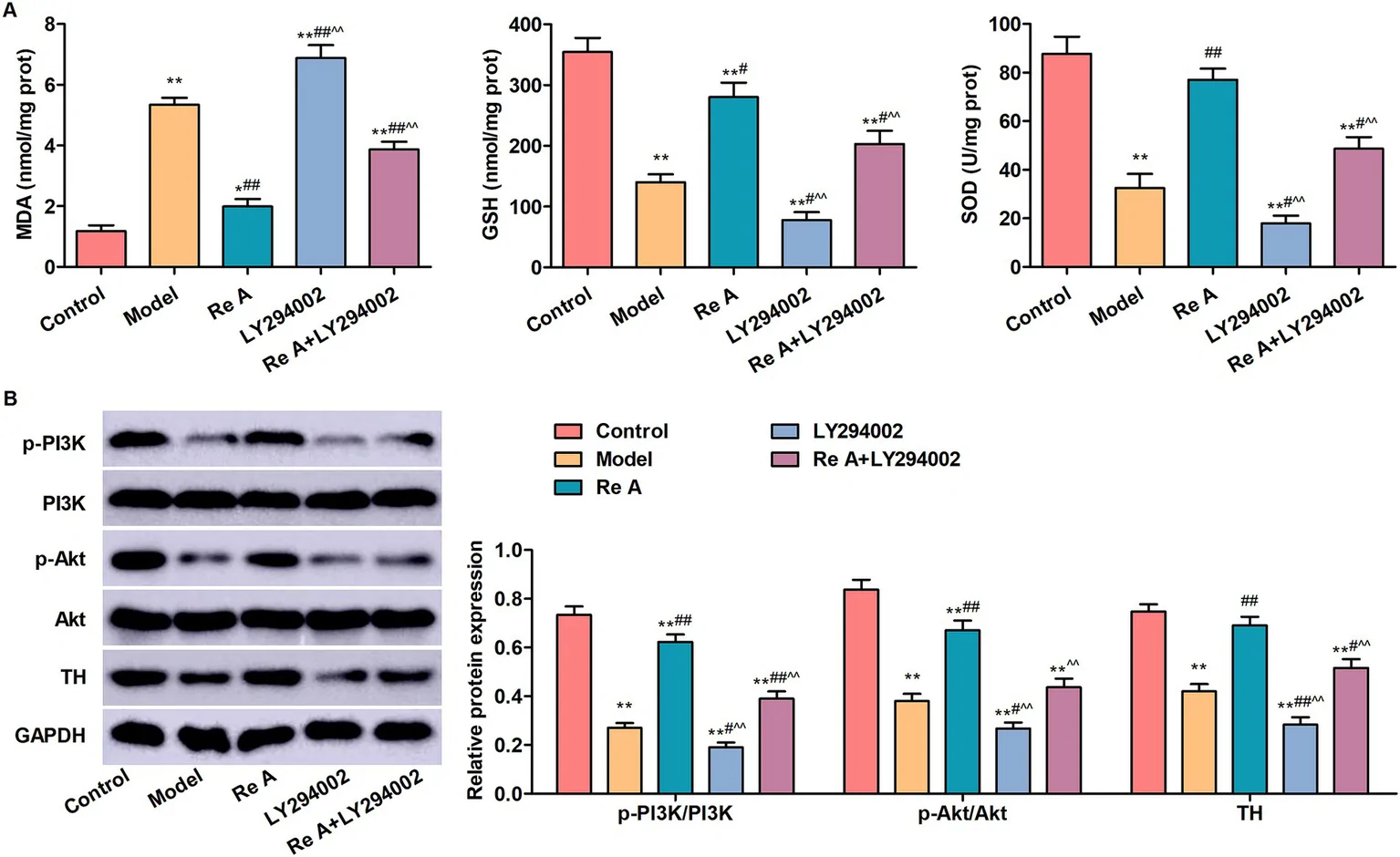
The PI3K inhibitor LY294002 reversed the protective effects of Re A in rotenone-treated PC12 cells. **(A)** MDA, GSH, and SOD levels in each group. **(B)** Protein expression of p-PI3K, PI3K, p-Akt, Akt, and TH detected by Western blot. Data are presented as mean ± SD (*n* = 6 per group for *in vivo* experiments; *n* = 3 independent experiments for *in vitro* assays). *p < 0.05, **p < 0.01 vs. Control group; #*p* < 0.05, ##*p* < 0.01 vs. Model group.

## 4 Discussion

The pathogenesis of Parkinson’s disease involves several interconnected processes, including oxidative stress, neuroinflammation, mitochondrial dysfunction, and dysregulated dopamine metabolism, which collectively contribute to neuronal oxidative damage and the eventual loss of dopaminergic neurons (24). For decades, deficiency of the neurotransmitter dopamine has been recognized as a core pathological feature underlying the motor symptoms of PD and remains a primary target for pharmacological intervention (25, 26). Current symptomatic therapies, predominantly based on levodopa replacement, do not halt or slow disease progression and are often associated with motor complications after long-term use (27, 28). Therefore, identifying agents with neuroprotective properties represents an important direction in PD research.

Rotenone, a mitochondrial complex I inhibitor, can be administered systemically to induce selective dopaminergic neuronal injury, α-synuclein aggregation, and motor deficits in animals, thereby reproducing key pathological hallmarks of PD (29, 30). However, the rotenone model also has limitations, including variability in lesion severity and peripheral toxicity. Although natural compounds have shown protective effects in rotenone-induced PD models, Re A has not been previously evaluated in this model. Using this well-established model, the present study aimed to evaluate the potential neuroprotective effects of the natural compound Rehmannioside A (Re A) and to preliminarily explore its underlying molecular mechanisms. Our study initially examined the protective effects of Re A at both cellular and animal levels. *In vitro*, pretreatment with Re A alleviated rotenone-induced loss of viability in PC12 cells. *In vivo*, rats subjected to rotenone-induced PD modeling exhibited characteristic impairments, including retarded weight gain and motor-behavioral deficits such as reduced motor coordination and endurance (rotarod test) and diminished spontaneous exploratory activity (open field test). Intervention with Re A attenuated these behavioral abnormalities to varying degrees, with more pronounced effects observed in the high-dose group. Consistent with these behavioral observations, HE staining of the substantia nigra pars compacta revealed that rotenone induced marked neuronal neuronal damage and pyknosis, which were visibly alleviated by ReA treatment. Immunohistochemical analysis further demonstrated that ReA dose-dependently increased the number of TH-positive neurons in the substantia nigra, suggesting preservation of dopaminergic neurons. Because Re A was administered before each rotenone injection throughout the modeling period, the present design primarily evaluates a neuroprotective/concomitant effect rather than a therapeutic effect on established degeneration. Together, these findings suggest that Re A may possess neuroprotective potential in this experimental model of PD.

Oxidative stress serves as a key driver of dopaminergic neuronal degeneration (24). Increased MDA and reduced GSH and SOD are consistent with the oxidative stress profile repeatedly reported in rotenone-induced PD models (29). Similar antioxidant protection has been observed with other natural compounds in PD models (31). In our study, the Re A-induced reversal of these markers was accompanied by PI3K/Akt activation and TH upregulation, which extends these previous antioxidant observations by linking them to a specific survival pathway. Our data showed that in the striatum of rotenone-modeled rats, the level of malondialdehyde (MDA), a terminal product of lipid peroxidation, was markedly increased, while the content of the endogenous antioxidant glutathione (GSH) and the activity of the critical antioxidant enzyme superoxide dismutase (SOD) were significantly reduced. These alterations collectively indicate a pronounced state of oxidative stress in the model. Previous studies suggest that pharmacological mitigation of oxidative stress can help protect dopaminergic neurons (32, 33). Concurrently, depletion of dopamine (DA) is a direct contributor to the motor symptoms of PD (34). Altered DA turnover, reflected by changes in DOPAC and HVA, has been described in PD models and may represent a compensatory response to dopaminergic injury (35). In the rotenone model used here, we observed a significant decrease in striatal DA levels, accompanied by elevated concentrations of its metabolites DOPAC and HVA. These changes are consistent with altered dopamine metabolism following dopaminergic injury, although a strict (DOPAC + HVA)/DA turnover ratio was not calculated in the present study. The Re A-induced increase in DA together with reduced DOPAC and HVA suggests a shift toward improved dopaminergic function. The parallel improvement in behavioral performance and restoration of biochemical parameters suggests that the protective effect of Re A may be related to its ability to alleviate oxidative stress and support dopaminergic system function.

To explore the potential molecular mechanisms underlying the effects of Re A, we focused on the PI3K/Akt signaling pathway, which is closely associated with cell survival, antioxidative defense, and neural function. Accumulating evidence indicates that activation of the PI3K/Akt pathway can attenuate neuronal damage, promote neuronal survival, and enhance cellular antioxidant capacity (31, 36). In rotenone-induced PD models, shikonin attenuated rotenone-induced neurotoxicity in SH-SY5Y cells via the IGF-1R/PI3K/Akt pathway (31), whereas cordycepin protected MPTP-treated mice by suppressing PI3K/Akt/mTOR- and MAPK-mediated neuroinflammation (11); PI3K/Akt has also been proposed as a therapeutic target in PD (12). These findings indicate that PI3K/Akt is a recurrent protective node in PD, but the upstream activators and the extent to which this pathway mediates antioxidant protection appear to differ among agents. To our knowledge, this is the first study to evaluate Re A in rotenone-induced PD models and to combine *in vivo* and *in vitro* evidence with LY294002 inhibition. The present work extends previous Re A studies, which mainly focused on vascular dementia, spinal cord injury, and ischemic cognitive impairment (17–19), by linking Re A treatment to improved motor behavior, preserved TH-positive neurons, restored striatal DA/DOPAC/HVA balance, and activated PI3K/Akt signaling. Tyrosine hydroxylase (TH), the rate-limiting enzyme in dopamine biosynthesis, is down-regulated in the early stages of PD (37, 38). Preservation of TH expression indicates maintenance of the dopaminergic phenotype and dopamine-synthesizing capacity, but TH immunohistochemistry alone does not distinguish true neuronal survival from TH downregulation in existing neurons. Our experimental results demonstrated that both PI3K/Akt pathway activity and TH protein expression were significantly suppressed in rotenone-injured PC12 cells and rat brain tissues. Re A treatment was associated with the reversal of these alterations: following Re A intervention, increased phosphorylation levels of the PI3K/Akt pathway and up-regulation of TH protein were observed. In line with previous reports, the PI3K/Akt pathway can positively regulate TH expression and protect it from oxidative damage (39, 40). PI3K/Akt signaling lies at the intersection of oxidative stress and mitochondrial function, and excessive oxidative stress can in turn suppress PI3K/Akt activity, forming a feed-forward cycle of mitochondrial dysfunction and dopaminergic degeneration. To further verify the involvement of this pathway, we employed the PI3K inhibitor LY294002 in PC12 cells. The results showed that LY294002 significantly reversed the antioxidant effects of Re A and attenuated its upregulation of p-PI3K, p-Akt, and TH expression, indicating that the protective effects of Re A are at least partly dependent on PI3K/Akt pathway activation. Therefore, our findings suggest that the neuroprotective effects of Re A may partly originate from its ability to activate the PI3K/Akt signaling pathway, thereby up-regulating TH expression and enhancing antioxidative capacity to preserve dopaminergic neuronal function. In addition, LY294002 is not fully specific for PI3K, and the present data therefore support, but do not definitively prove, PI3K/Akt dependence. Pharmacological inhibition and genetic knockdown are complementary approaches: pharmacological inhibition allows rapid and reversible modulation of kinase activity but carries a risk of off-target effects, whereas genetic knockdown offers greater target specificity and would provide stronger evidence for a causal role of PI3K/Akt. In this study, genetic knockdown was not performed, and future studies using siRNA or shRNA targeting Akt or the PI3K p110 subunit will be necessary to establish causality. However, PC12 cells are an undifferentiated rat pheochromocytoma cell line and were used strictly as a high-throughput surrogate screening platform to interrogate fundamental intracellular signaling cascades (e.g., PI3K/Akt phosphorylation), rather than to model mature neuronal cytoarchitecture, synaptic physiology, or human dopaminergic metabolic dynamics. The *in vitro* findings may therefore not fully recapitulate the vulnerability of substantia nigra dopaminergic neurons *in vivo*. Primary midbrain dopaminergic cultures or human iPSC-derived dopaminergic neurons would provide a more physiologically relevant validation and will be an important direction for future studies.

In this study, we therefore interpret PI3K/Akt as an important mediator, but not necessarily the sole initial trigger, of Re A-mediated antioxidant protection. A limitation of the present study is that we did not directly measure Re A concentrations in brain tissue or CSF, and therefore cannot formally establish that orally administered Re A crosses the blood–brain barrier. The observed changes in brain PI3K/Akt signaling and TH expression may reflect several possibilities: Re A or its active metabolites may reach the brain and act directly on dopaminergic neurons; Re A may act peripherally to reduce oxidative stress and inflammation, indirectly protecting the nigrostriatal system; or both mechanisms may contribute. The present data cannot distinguish between these possibilities. Future pharmacokinetic studies measuring Re A and its metabolites in plasma and brain tissue, together with blood–brain barrier permeability assays, will be necessary to determine the extent of its brain penetration.

Levodopa + Carbidopa was selected as the positive control because it represents the current standard symptomatic therapy for PD and allows validation of the rotenone model and the sensitivity of the behavioral and biochemical readouts (41). However, levodopa acts primarily as a metabolic dopamine precursor and restores dopaminergic transmission through dopamine replacement rather than through direct modulation of PI3K/Akt phosphorylation. Therefore, the comparable effects of high-dose Re A and levodopa on p-PI3K, p-Akt, and TH expression should not be interpreted as evidence that both agents act through the same mechanism. Rather, these findings suggest that Re A and levodopa may converge on similar downstream endpoints—preserved TH expression and dopaminergic function—through distinct upstream mechanisms. This distinction supports the interpretation that Re A may exert neuroprotective effects via PI3K/Akt-dependent signaling, whereas levodopa primarily provides symptomatic dopamine replacement.

Rehmannioside A is an iridoid glycoside component found in the traditional Chinese herb Rehmanniae Radix. Compounds of this class have been reported to possess various biological activities, including anti-inflammatory and antioxidant effects (42). Previous studies have reported protective effects of Re A in vascular dementia, spinal cord injury, and ischemic cognitive impairment, mainly through antioxidant, anti-inflammatory, and PI3K/Akt-related mechanisms (17–19). The present study extends these findings to PD by showing that Re A improves motor behavior, preserves TH-positive neurons, restores striatal DA/DOPAC/HVA balance, and activates PI3K/Akt signaling in rotenone-induced models. In summary, our research indicates that Rehmannioside A can alleviate oxidative stress, improve dopaminergic system function, and mitigate motor deficits in rotenone-induced PD cell and animal models. These findings provide a preliminary basis for further preclinical evaluation of Re A.

## Data Availability

The original contributions presented in the study are included in the article/supplementary material, further inquiries can be directed to the corresponding author/s.
